# Preparation, Bioactivities and Applications in Food Industry of Chitosan-Based Maillard Products: A Review

**DOI:** 10.3390/molecules26010166

**Published:** 2020-12-31

**Authors:** Huijuan Yang, Yuyu Zhang, Fang Zhou, Juanjuan Guo, Jiajie Tang, Yanqing Han, Zhanming Li, Caili Fu

**Affiliations:** 1 College of Standardization, China Jiliang University, Hangzhou 310018, China; huijuanyang2012@gmail.com; 2 Beijing Key Laboratory of Flavor Chemistry, Beijing Technology and Business University (BTBU), Beijing 100048, China; caili.fu@nusri.cn; 3 Fujian University Key Laboratory of Biotechnology for Offshore Resources, Quanzhou Normal University, Quanzhou 362000, China; fang.zhou@nusri.cn (F.Z.); gjjfst15@163.com (J.G.); 4 National University of Singapore (Suzhou) Research Institute, 377 Lin Quan Street, Suzhou Industrial Park, Suzhou 215123, China; tangjiajie1998@yeah.net (J.T.); yanqing.han@nusri.cn (Y.H.); lizhanming@just.edu.cn (Z.L.)

**Keywords:** chitosan, Maillard reaction, derivatives, application

## Abstract

Chitosan, a biopolymer possessing numerous interesting bioactivities and excellent technological properties, has received great attention from scientists in different fields including the food industry, pharmacy, medicine, and environmental fields. A series of recent studies have reported exciting results about improvement of the properties of chitosan using the Maillard reaction. However, there is a lack of a systemic review about the preparation, bioactivities and applications in food industry of chitosan-based Maillard reaction products (CMRPs). The presence of free amino groups in chitosan allows it to acquire some stronger or new functional properties via the Maillard reaction. The present review aims to focus on the current research status of synthesis, optimization and structural identification of CMRPs. The applications of CMRPs in the food industry are also discussed according to their biological and technological properties such as antioxidant, antimicrobial activities and inducing conformational changes of allergens in food. Some promising directions for future research are proposed in this review, aiming to provide theoretical guidance for the further development of chitosan and its derivatives.

## 1. Introduction

Chitosan, a biopolymer, possesses different bioactive properties such as antimicrobial, anti-inflammatory as well as antioxidant properties, which make it an interesting compound for applications in different fields such as the food industry, pharmacy, medicine and environmental fields [1,2]. As a kind of polysaccharide, chitosan products have received considerable attention in recent years for their favorable biological properties, non-toxicity, biocompatibility and biodegradability [3,4,5,6].

Among most studies, it has been reported that chitosan also has antimicrobial and antioxidant activities which promote its use in the fields of food protection and the pharmaceutical industry in particular [7]. In addition to effectively enhancing the shelf life of food ingredients, preventing oxidative damages and inhibiting food-borne pathogens development, it can also increase drugs’ solubility and retention in the site where they are applied [2,8,9,10,11]. However, chitosan has certain limitations and not all chitosans display optimal properties, and research has shown that chitosan with a poor water-solubility at neutral or basic pH and therefore may have limited applications [12,13]. Moreover, the functional characteristics of chitosan can be influenced by many factors, such as the degree of deacetylation, the molecular weight and its source [14,15].

Several strategies have been developed in attempts to eliminate these obstacles (i.e., the poor solubility and low degree of deacetylation) in order to improve the properties [16,17]. Previous studies have shown that the properties of chitosan can be improved by the use of chemical and physical modifications such as heat treatment and the acylation reactions to prepare new derivatives [18,19]. However, these modifications cannot be widely used in food and other fields because of limited and unstable control of these chemical and physical methods [13]. Moreover, studies have reported that chitosan derivatives prepared through acid hydrolysis and enzymatic hydrolysis encounter have activity stability problems and high cost, respectively [13,20,21]. Therefore, the development of efficient and effective strategies for the production of optimum chitosan-based derivatives is very essential.

The Maillard reaction, also called non-enzymatic browning, is recognized as a mild browning reaction caused by the amino groups of amino acids in the presence of the carbonyl groups of sugars [13]. It is also recognized that the preparation of materials with single or multiple functional groups can promote the biological properties of many bioactive compounds [22,23]. With the presence of amino groups, chitosan can be modified by the Maillard reaction by reacting it with sugars and this changes its physical and biological properties such as antimicrobial activity, which can enhance its applications in many fields [24,25]. It was also reported that chitosan-based Maillard reaction products (CMRPs) reacted with monosaccharides exhibited improved solubility and significantly higher antioxidant activity than chitosan itself [26]. Another study also stated that the degree of deacetylation of chitosan decreased by approximately 12% after reacting with glucosamine via a Maillard reaction [25]. In conclusion, the Maillard reaction has the ability to improve the physical and biological characteristics of chitosan. Moreover, CMRPs may be promising substances for their applications in food, pharmacy, medicine and environmental fields.

Previous reviews were mainly focused on the characteristics, biological properties and applications of chitin and chitosan [1,4,27,28,29]. However, there is a lack of a systemic review about preparation, bioactivities and applications of CMRPs. Therefore, the current research status of synthesis, structural identification and biological properties of CMRPs were reviewed in this study. Moreover, the applications of CMRPs and some suggestions for future research have also been proposed in this review.

## 2. Chitosan

Chitosan is mainly present in the exoskeleton of crustaceans and the cuticles of insects [30,31]. It consists of randomly distributed d-glucosamine and *N*-acetyl-d-glucosamine linked by β-(1-4)-glycosidic bonds and is prepared by *N*-deacetylation of chitin, the second most abundant biopolymer in Nature after cellulose [2,32]. As a natural and renewable resource, chitosan has many applications in fields such as medicine, agriculture and environmental protection due to its properties such as biocompatibility and biodegradability [33,34]. The applications of chitosan in different fields are correlated with the physiochemical and biological properties it offers [2,9,35].

### 2.1. Physicochemical Characteristics

One of the basic indexes of chitosan is its molecular weight, which is often used as a basic standard to evaluate its quality, the reason being that low molecular weight chitosans can be used as antibacterial, antioxidant and anti-tumor substances [36]. Research has also shown that chitosan with a medium molecular weight exhibits a higher anti-cholesterol activity compared with chitosans with a high molecular weight [37]. The molecular weight of chitosan depends mainly on the degree of deacetylation of chitin and can be calculated according to the formulas reported by [38,39,40].

Degree of deacetylation is also an important index to evaluate the physical, chemical and biological properties of chitosan, such as degradation and cell response [35,36]. The degree of deacetylation of chitosan is determined by the reaction between the polymer and concentrated sodium or potassium hydroxide solutions. During this process, high temperatures (100 °C or higher), long reaction times (30 min or longer) and higher NaOH/KOH concentrations are usually required because the removal of acetyl group from the chitin can be promoted by more optimized conditions [14,35,41]. In addition to the influence of different reaction conditions, the degree of deacetylation of chitosan is also correlated with the different biological sources of chitosan [14]. The degree of deacetylation is commonly calculated by many methods such as the Fourier transform infrared spectroscopy (FTIR) method [36,42] and the modified linear potentiometric titration method [43].

The solubility of chitosan is influenced by many factors. At neutral and basic pH values, chitosan has a poor water solubility which limits its uses in many fields [44,45]. Compared with other neutral or negatively charged polysaccharides, however, chitosan has a unique cationic nature at low pH value which increases its retention at the site of application, which has garnered a lot of attention for agricultural, medical and pharmaceutical applications [2,9,46]. The solubility of chitosan also depends on its molecular weight and degree of deacetylation. Low molecular weight and high degree of deacetylation of chitosan result in high solubility [47]. The method for measuring the solubility of chitosan was described in previous research [36].

Viscosity is another important indicator to evaluate the quality of chitosan. The viscosity of chitosan increases with the increase of molecular weight and degree of deacetylation [35]. The viscosity of chitosan is also affected by other factors such as the temperature, and it is reported that the viscosity of chitosan is stable at 4 °C. Chitosan with high viscosity can more easily form chitosan membranes which perform better in the pharmaceutical excipient field [40]. The viscosity of chitosan can be determined by dissolving the prepared chitosan in 0.5 M acetic acid 0.5 M sodium acetate buffer at 25 °C and following the detailed description reported in [48].

### 2.2. Biological Activities

Many studies have shown that chitosan possesses strong antimicrobial activity against a wide range of microorganisms, such as fungi (molds and yeasts), Gram-positive and Gram-negative bacteria [8,11]. Some studies have reported that chitosan is considered to possess stronger antibacterial activity against Gram-negative bacteria compared with Gram-positive bacteria, although there is some controversy regarding this fact as the opposite has also been stated by other authors [49,50,51]. There are several factors that affect the antibacterial and antifungal activities of chitosan. Variation in the degree of deacetylation, molecular weight, solubility and types of chitosan can cause varied effects on chitosans’ antimicrobial properties. As the degree of deacetylation increases, the antimicrobial activity is also augmented which may due to the increased number of amino groups on chitosan [52,53]. A study has shown that low molecular weight chitosan has greater antimicrobial activity than that with high molecular weight [54], which may due to the fact that low molecular weight chitosan has better solubility properties, as mentioned before. Chitosan coming from different sources may also hold different antibacterial activity, and marine chitosan is found to have a better activity than fungal chitosan [55]. Previous studies also found that the peptide obtained from tuna backbone protein via enzymatic hydrolysis exhibited the highest antioxidant activity compared to other hydrolysates [56].

Chitosan has antioxidant activity because it can react with free radicals [57]. Studies have measured the antioxidant efficiency of chitosan based on its scavenging activity in the superoxide assay [58]. Another study evaluated the antioxidant activity of chitosan from the blowfly larva through the results of DPPH scavenging assays and found that blowfly chitosan might be used as a natural antioxidant [59]. The antioxidant effect of chitosan with different physicochemical characteristics was also studied. A previous study used chitosans with different molecular weight (30, 90, and 120 kDa) to investigate their antioxidant activity, and the results showed that the chitosan of 30 kDa molecular weight exhibited the highest antioxidant activity [60]. In addition, studies showed that chitosan with high degree of deacetylation exhibited higher radical scavenging effects [61,62].

### 2.3. Other Applications of Chitosan

The physicochemical characteristics and biological activities of chitosan make it useful in many different applications. Chitosan can promote wound healing and prevent bacterial infections because of its antimicrobial and film-forming activities [51]. A study showed that chitosan membranes had the ability to decrease wound healing time [63]. In addition, chitosan can also form biodegradable films which can be used for food packaging and drug delivery applications and many reviews have focused on the literature regarding its practical application in food packaging [7,64,65]. In addition, anti-cancer drugs encapsulated in chitosan-based nanocarriers showed increased solubility and enhanced permeability and retention [9,66]. Moreover, chitosan has attracted a lot of scientists’ interest in the water treatment field due to its biodegradability and non-toxicity [5], and researchers also reported that chitosan-based biosorbents can remove toxic metal ions and many kinds of dyes from water [67,68].

## 3. Preparation and Identification of Chitosan-Based Maillard Products

### 3.1. The Preparation of CMRPs

The applications of chitosan are limited because of its poor solubility at neutral or basic pH, as mentioned previously. Research also found that this could be attributed to high crystallinity in its inner structure due to hydrogen bonds and acetamido groups [69]. However, chitosan reacted with different kinds of sugars via Millard reaction can produce different CMRPs with improved properties, which have attracted the attention of researchers because of their potential activities and applications in many fields [24,25]. Moreover, the preparation methods of CMRPs of different studies might have some slight differences.

Hamdi et al. stated that after dissolving chitosan in an aqueous solution of acetic acid, sugars (glucose, fructose, arabinose and xylose) were added under gentle stirring for 24 h at 25 °C. After a few other procedures, the well-dried chitosan films were then heated for 24 h at approximatively 90 °C and an 30% relative humidity for Millard reaction induction [26]. Hafsa et al. used different concentrations of galactose, and added them into a chitosan solution under gentle stirring (the time and temperature were not specified). Then the mixtures were put into tubes for heating at 100 °C for 3 h in a water bath to conduct the chitosan-galactose Maillard reaction [70].

Zhu et al. reported that CMRPs prepared from chitosan and xylose showed increased chelating power as the heating time increased (in a water-bath for up to 8 h) [71]. From these studies, we find that the Maillard reaction under similar temperatures differed in their reaction time (24 h, 8 h or 3 h) which may be due to the differences in the sources of the chitosan and sugars used in these studies. It is also recognized that the structure and physicochemical properties of compounds influences their biological properties [22,28].

Some researchers didn’t adopt the water-bath method for heating. Nooshkam et al. added inulin into a chitosan solution (the ratio of chitosan to inulin was 3:1) and then the mixed solution was stirred for 30 min. Afterwards, the mixed solutions were autoclaved for 15 min to form the corresponding Maillard derivatives [23]. However, Braber et al. mixed glucosamine and chitosan in an orbital shaker in an oven at 65 °C (without using the water bath method) to induce the Maillard reaction and the complete conversion to CMRPs from the original chitosan occurred after 48 h of heating [25]. Kosarju et al. sealed mixed chitosan and glucose in metal cans and heated them at 98 °C up to 2 h, and the indices related to the extent of Maillard reaction were thereby increased [71]. It could be concluded that using an autoclave may be a very efficient and time-saving approach to perform the Maillard reaction.

In some studies, ultrasonic waves have also been used to assist the conventional heating methods such as water-bath heating for the improvement of CMRPs’ properties [13,72]. A previous study found that the use of high intensity ultrasonic waves (400 W, 28 kHz, 17.83 w/cm^2^)-assisted water-bath heating (at 80 °C for 1–8 h) could change the physicochemical properties of the resulting material (fructose was mixed with chitosan) such as increased solubility, and biological functionality such as antioxidant and antibacterial activities [13].

In conclusion, Maillard reactions under different conditions (such as heating time and temperatures) may vary in the preparations of CMRPs. Table 1 compares the preparation methods of CMRPs from previous studies and summarizes the differences between them and their performance. Future studies may focus on some efficient methods (such as the used of autoclave) and combined methods (such as UV-assisted methods) to produce the CMRPs that may promote their industrial use.

### 3.2. Methods Used for the Identification of CMRP Formation

#### 3.2.1. UV Absorbance and Fluorescence Intensity

During the formation of CMRPs, the extent of the Maillard reaction can be observed by the absorbance at 294 nm (the intermediates are colorless) and at 420 nm (the final products are brown) using an ultraviolet-visible diode array spectrophotometer for the intermediate and the final stages, respectively [23,79,80]. Some studies also use the ratio of A_294_/A_420_ to evaluate the extent of the Maillard reaction. The increased A_294_/A_420_ indicates the intermediate stage, while the decreased A_294_/A_420_ indicates the final stage of the reaction [16,74]. During the Maillard reaction, fluorescent compounds (the intermediates) are also developed and recognized as the precursors of the brown compounds [3,81,82]. The fluorescence intensity was measured at an excitation wavelength of 346 nm and emission wavelength of 415 nm on a fluorescence spectrophotometer [83]. Studies showed that the increased fluorescence intensity of the mixtures suggesting the CMRPs formation and decreased fluorescence ability observed during the reaction suggesting the final stage of the formation of CMRPs [70,74].

#### 3.2.2. Fourier Transform Infrared (FTIR) Analysis

FTIR spectrometry is used for the analysis of the chemical groups formed by Maillard reaction and examination of the possible interaction mechanisms between the substances at the absorbance mode from 4000 to 500 cm^−1^ [16]. The FTIR spectra of the conjugates should be compared with the corresponding initial chitosan to examine the differences [3]. Researches showed that the characteristic absorption bands can be observed around 3340 cm^−1^ (attributed to −NH and −OH) and 2930 cm^−1^ (attributed to −CH) [13]. The peaks at around 1650, 1560 and 1384 cm^−1^ were assigned to amide I, II, and III absorption bands of chitosan, respectively. The bands at 1420 cm^−1^ and 1375 cm^−1^ were attributed to the deformation of the −CH_2_ and −CH_3_ groups, respectively [77,84,85]. The changes of bands and peaks compared with the initial chitosan may indicate that the sugars have been attached to chitosan. Studies have reported that a decrease in the band at 1560 cm^−1^ was observed with the time of reaction which suggested that the amines II were converted into amines III [16,86]. Another study showed that the peak at 3345 cm^−1^ became less wider indicating the interaction between chitosan and carbonyl group of galactose [70].

#### 3.2.3. High Performance Liquid Chromatography-Size Exclusion Chromatography Analysis (HPLC-SEC)

The chromatograms can be obtained from the HPLC-SEC analysis of the initial chitosan and the CMRPs to examine and compare the differences before and after the reaction. The average molecular weight can also be obtained after the chitosan and CMRPs were submitted to HPLC-SEC analysis [21,25,74]. Study showed that it could be easily found from the chromatograms that the peak corresponding to the Chitosan-galactose is wider than that of the chitosan alone [16].

#### 3.2.4. Color Measurements

Color measurement by a colorimeter is also used to evaluate the stages of the Maillard reaction and represents the generation of CMRPs. The color parameters (L*, a* and b*) are used to determine the degree of browning, and the decreased values of L* and a*, and increased values of b* indicate the browning of the mixtures [13,73,76].

#### 3.2.5. Colloid Titration Method

A decrease in degree of deacetylation of chitosan derivatives could be observed during the Maillard reaction. The colloid titration method is the most useful method to determine the degree of deacetylation [87,88].

#### 3.2.6. Proton Nuclear Magnetic Resonance (^1^H-NMR)

In order to confirm the of the sugars’ units in the chitosan molecules, ^1^H-NMR can be used to observe the differences between the native chitosan and its derivatives, and their structural changes inferred from the spectra [74]. A study using ^1^H-NMR analysis reported that signals appearing at 2.09 ppm, corresponding to −CH_2_ linked to the amino group, suggesting a displacement of the –N=CH– group (a Schiff base, an intermediate of the Maillard reaction) [16].

Some methods used in previous articles are also very useful in identifying the formation of CMRPs and can be used as auxiliary assays accompanying with other methods. Differential scanning calorimetric (DSC) measurement is used to present the peak temperatures shift of the chitosan and its CMRPs. The DSC data could be used to evaluate the degradation process and corroborate the results obtained form other methods [13,89,90]. Transmission electron microscope (TEM) observation is used for the detailed shape of samples, and X-ray diffraction (XRD) assay can be used to determine the packing structures of samples [72]. It has been reported that the decrease of pH value of the mixtures happens during the Maillard reaction between reducing sugars and amino groups, which can also be used as an auxiliary means [91,92].

## 4. Biological Activities of CMRPs

**Figure 1:**
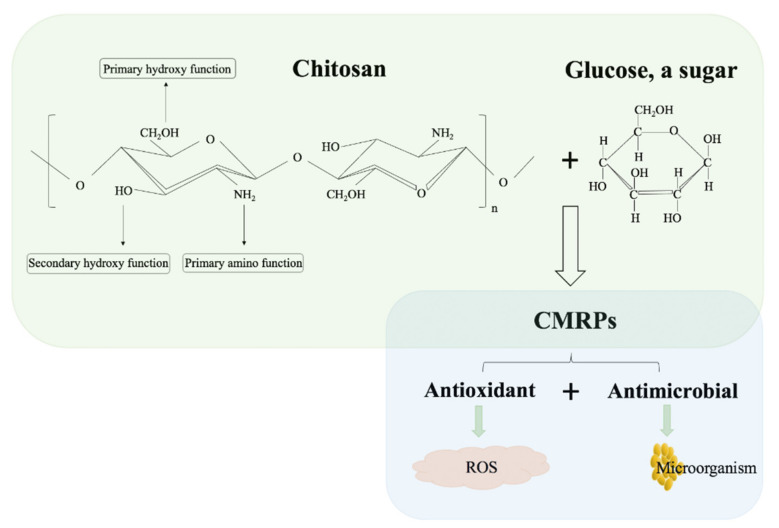
The chemical structure of chitosan and properties of CMRPs [16,25,35].

Among all biological properties of chitosan, the antioxidant and antimicrobial activities are of great importance (Figure 1) [4,93]. Producing new materials with single or multiple functionalities can promote the biological properties of many bioactive compounds [22,23]. Several researches have reported that the chitosan-based derivatives formed by Maillard reactions exhibited improved biological properties compared with the initial chitosan [25,73,75]. 

### 4.1. Enhanced Antimicrobial Activity

Results illustrated that the Maillard reaction could enhance the antimicrobial activity of chitosan and the antimicrobial activity of CMRPs may be attributed to several antimicrobial mechanisms, such as their high surface activity and interference with the microorganisms’ membranes [94].

Liang et al. suggested that the conjugate with polylysine and chitosan (1:1) exhibited the strongest anti-fungal activity compared with the chitosan alone indicated by the reduction of the inhibition zone area, and the diameter of the inhibition zone of the conjugates against beer yeast decreased as the dry-heating time was extended. Moreover, the damage of the outer and inner membranes in *E. coli* (Gram-negative bacteria) and *S. aureus* (Gram-positive bacteria) demonstrated the stronger antibacterial activity of CMPRs [3]. Zhang et al. also reported that chitosan-fructose Maillard reaction products also showed higher antibacterial activity against *S. aureus* and *E. coli* than the initial chitosan because the CMPRs disrupted the membrane integrity of the microorganism which inactivated them. Moreover, the chitosan-fructose Maillard reaction products interacted more effectively with the bacterial cells may due to their positively charged status [13].

In addition, there are some other factors that can affect the antimicrobial effects of CMRPs. Nooshkam et al. found that the antimicrobial effects varied from group to group which may due to the pH values of the solutions and the levels of browning intensity. In that research, the chitosan–inulin Maillard reaction products with lower browning intensity formed at low pH values (5.0 and 5.5) showed higher inhibitory effects than that of higher groups [23]. Li et al. found that the antimicrobial activities differed depending on the chitosan molecular weight. The application of prepared CMRPs formed from maltose and low molecular weight chitosan on fresh-cut *Typha latifolia* L. effectively delayed the increasement of aerobic mesophilic bacteria for 3 days and put the populations of lactic acid bacteria under control during the whole storage period which was longer than the storage time in the group treated with maltose or low molecular weight chitosan only [73].

### 4.2. Enhanced Antioxidant Activity

In order to investigate the antioxidant activity of the CMRPs, some in vitro antioxidant test methods are often used, such as the 1,1-diphenyl-2-picrylhydrazyl (DPPH) radical scavenging and reducing power assay. The DPPH radical scavenging activity method is also often used to test the anti-oxidant activity of CMRPs. DPPH radicals have been widely used to evaluate the free radical scavenger and hydrogen donation activity if substances [83,88]. The reducing power could be attributed to the primary phase or final stage compound formation during the process of Maillard reaction [95]. The enhanced antioxidant activity is not only attributed to the more active amino groups reacting with DPPH radicals, but also attributed to the attachment of sugars and Schiff-bases on the chitosan, which enhances the ability to scavenge DPPH radicals [96].

Hamdi et al. adopted DPPH radical scavenging and reducing power assays to detect the free radical quenching and iron reduction ability, respectively. The antioxidative mechanism could be attributed to the effects of radicals scavenging and metal chelation [97]. Data in this work revealed that the chitosan-aldopentoses Maillard reaction crosslinked films’ antioxidant activity was remarkably higher than that of the chitosan films only [26]. Koaraju et al. reported that the CMRPs made from acid-soluble chitosan reacted with glucose for 30 min at pH 4.9 showed higher antioxidant activity as indicated by ferric reducing antioxidant power (FRAP) and oxygen radical absorbance capacity (ORAC) assays [71]. In addition, Badano et al. stated that chitosan-lactose derivatives showed an increased ABTS radical cation (ABTS^+^) scavenging activity compared to that of chitosan alone. The results also showed that the derivatives also had an improved OH^·^ scavenging effect and a significant O_2_^−^ scavenging activity which demonstrated the antioxidant activity of chitosan-lactose Maillard reaction products [77].

There are some factors that may also significantly influence the antioxidant activity of the CMRPs, such as reaction time, pH values and reaction degree.

Tran et al. found that the water-soluble chitosan–glucose derivatives produced by Maillard reactions exhibited higher antioxidant activity than the initial water-soluble chitosan, and the antioxidant ability of derivatives increased as the heating time and temperature increased during the Maillard reaction [74]. Zhu et al. also found that the antioxidant properties of CMRPs prepared from chitosan and xylose is also increased as the reaction time was prolonged. The scavenging activity of CMRPs, however, became lower when the reaction time increased to 8 h [75].

The chitosan–inulin Maillard reaction products with lower browning intensity obtained at low pH values showed increased antimicrobial effects, whereas Nooshkam et al. also found that the conjugates with high browning intensity, which was obtained at high pH values, exhibited higher DPPH radical scavenging activity than other conjugates. In conclusion, this study demonstrated that the CMRPs prepared from chitosan and inulin could be used as bioactive derivatives with better antimicrobial and antioxidant properties [23].

In conclusion, those results showed that the addition of CMRPs imparted a higher antioxidant activity to the products and CMRPs could be used as promising additives against food oxidation.

## 5. Applications of CMRPs

The Maillard reaction is one of the most important reactions which takes place during food processing without the addition of catalysts or organic solvents [98,99]. Maillard reaction-induced modifications of chitosan could be a most promising approach for food, medicine, pharmacy and medicine applications because of the advantages mentioned above. Many scientists have investigated the applications of CMRPs’ which may be used as the directions for further research correlated with the field of CMRPs in the future. Table 2 also summarizes some applications of CMRPs reported in previous studies.

### 5.1. Application in Food Storage

Several researches have suggested that CMRPs might be used as additives and promising vegetable preservatives in the food industry. Hafsa et al. prepared CMRPs based on chitosan and 1%, 1.5% and 2% (*w*/*v*) of galactose and found that CMRPs produced with 2% of galactose, exhibited better antioxidant activities in apple juice than chitosan alone [70]. Li et al. evaluated the CMRPs prepared from maltose and different molecular weight chitosans and their effects on the preservation of fresh-cut *Typha latifolia* L. (TLL). Data suggested that high molecular weight chitosan-based derivatives showed better effects on inhibiting the discoloration and ascorbic acid content of fresh-cut TLL during storage, as compared to chitosan only [73].

Zhu et al. incorporated CMRPs prepared from chitosan and xylose into semi-dried noodles for 6 h at room temperature which resulted in an extended shelf-life for more than 7 days compared with the control group. Moreover, the addition of CMRPs effectively limited the discoloration and darkening of the semi-dried noodles [75]. Another previous study by Huang et al. also reported that the addition of CMRPs (also prepared from chitosan and xylose) to fresh noodles resulted in longer shelf-life for 14 days when stored at 4 °C, while the group with the addition of chitosan alone exhibited a shelf-life of approximately 7 days [103].

CMRPs can also be used in meat products. Amaral et al. concluded that the derivatives formed by chitosan and glucose via Maillard reactions extended the shelf-life of fresh goat sausages stored under chilled conditions because the CMRPs-treated samples showed inhibited microbial growth and the CMRPs also made the fresh goat sausages firmer and more stable than the control samples [101]. Another study showed that fresh pork samples dipped also in chitosan–glucose Maillard reaction products just for 10 min and stored at 4 °C exhibited lower thiobarbituric acid-reactive substances (TBARS) values (indicators of lipid oxidation) and microbial counts. Moreover, the study also reported that the treatment of CMRPs had no negative influences on the sensory qualities of the pork samples [102].

Chitosan with a certain polymerization degree, often generated by enzymatic or chemical hydrolysis, is known as a chitooligosaccharide (COS) [103]. COS has attracted a lot of attention due to its good water-solubility, and its Maillard reaction products have also been investigated and exhibit potential applications due to their improved biological properties. Yu et al. reported that CMRPs prepared from COS and lysine, retarded the surface darkening of fresh-cut kiwifruit and inhibited the microbial growth of samples [104]. Zheng et al. studied the CMRPs prepared from COS and nisin, and found that the conjugates had a better effect on preservation of *Collichthys niveatus*, a marine species with delicious flavor [99]. Yan et al. also found that the CMRPs (prepared from COS and glycine) -treated fruit juices samples exhibited a higher antioxidant capacity [105].

### 5.2. Application in Health Foods or Medicine

Type 2 diabetes caused by insulin resistance may also cause many complications to patients, such as cardiovascular disease [106]. Approaches that have the ability to retard the activities of α-amylase and α-glucosidase may be used as an effective therapy for managing type 2 diabetes. In this context, Tran et al. found in their research that chitosan-glucose derivatives produced via Maillard reactions exhibited higher anti-α-amylase and anti-α-glucosidase activities [74].

The urgent development of safe and effective therapeutic strategies for the prevention of obesity is essential [107]. The chitosan oligosaccharide-nisin conjugates formed by Maillard reaction showed protective effects against obesity induced by high-fat diet suggested by the alleviated microphage accumulation in mice liver and obesity-induced gut dysbiosis [99].

Tropomyosin is the main allergen in shellfish that causes many severe food allergy cases around the world [108]. Fu et al. found that the Maillard reaction between chitosan-oligosaccharide and tropomyosin could play an important role in tropomyosin allergenicity. In fact, the reported data showed that Maillard reaction with the sugars modified both tropomyosin amino acid residues and the molecule secondary structure features. These structural changes significantly reduced the ability of specific IgE antibodies, contained in the sera of allergic subjects, to recognize and bind the allergenic protein [109].

Therefore, the CMRPs might be attractive candidates for future use in the medicine or health food fields.

## 6. Conclusions and Future Research

Chitosan reacts with sugars via Maillard reactions to form some derivatives that have exhibited improved properties which may promote their applications in different fields, such as food and medicine. This review not only discussed the properties of chitosan, but also the preparation, identification, enhanced properties and potential applications of CMRPs. However, there are some limitations in the field of CMRPs that still need future investigation. Chitosan reacted with different sugars via Maillard reactions may produce many bioactive compounds under different reaction conditions. Thus, more studies are needed to investigate these compounds formed during Maillard reactions and evaluate their properties. Moreover, CMRPs have improved antimicrobial activities which may due to their positively charged status and effective interaction with the negative residual charge on the surface of bacterial cells [13]. However, the underlying molecular mechanism also need to be investigated and clarified. Finally, while the future mass market applications of CMRPs are of great importance, not so many studies have been done and not all CMRPs with enhanced biological properties have been applied in food, medicine or any other fields to evaluate their practical applications at present. Therefore, further studies are needed to focus on that aspect to confirm their effectiveness.

## Figures and Tables

**Table 1:** An overview of methods for preparing CMRPs.

Materials Reacted with Chitosan	Stirring Methods	Heating Methods	Performance	References
Polylysine	Not specified	Dry-heated with different periods (0, 3, 6, 9 and 12 h) in a desiccator (80 °C, relative humidity: 79%) containing saturated potassium bromide solution at the bottom	Extension in the dry-heating time reduced the antibacterial activity of conjugates	[3]
Fructose	Not specified	Put in a test tube subjected for ultrasound-assisted water bath at 80 °C for 8 h	DPPH free radical scavenging capacity from UA heating were higher than those from water-bath heating alone	[13]
Glucose	Under shaking at 40 °C, 60 °C or 80 °C 100 rpm for different reaction time		The best conditions were 60 °C and 32 h of reaction.	[16]
Inulin	Stirred for 30 min	Autoclaved for 15 min	The reaction time was reduced by using autoclave	[23]
Glucosamine hydrochloride	Under shaking at 65 °C for 48 h in an oven		After 48 h of heating, the complete CMRPs were obtained from the original chitosan	[25]
Glucose, fructose, arabinose and xylose	Gentle stirred for 24 h at 25 °C	Heated for 24 h in an oven at approximatively 90 °C and an 30% relative humidity	Heat treatment did not affect their mechanical parameters, while decrease their hydrophobicity	[26]
Galactose	Stirring (other were not specified)	Put inside a test tubes and for heated for 3 h at 100 °C in water bath	Heated chitosan with 2% (*w*/*v*) of galactose for 3 h was the most efficiency concentration	[70]
Glucose	Not specified	Sealed in metal cans and heated at 98 °C up to 2 h	Increased all of these indices of the extent of Maillard reaction	[71]
Maltose	Not specified	Heated for 6 h at 100 °C in water bath	Prolonged heating time increased the DPPH free radical scavenging activity of CMRPs	[73]
Glucose	Not specified	Heated at 100 °C (using a water bath) or 121 °C (using an autoclave) for 1–4 h	Heating temperature at 121 °C had a higher efficiency than 100 °C in the formation of intermediates	[74]
Xylose	Not specified	Heated at 80 °C in water bath for up to 8 h	Higher chelating power was observed as the increase of heating time	[75]
Glucose and cellobiose	Stirred for 30 min at 25 °C	Dried in an oven at 50 °C for about 20 h	Not specified	[76]
Lactose	Under shaking at 70 °C, 120 rpm for 96 h		The reduction in the molecular weight	[77]
Corn protein hydrolysate	Not specified	Incubated for 48 h in a desiccator (60 °C, relative humidity: 79%) containing saturated potassium bromide solution at the bottom	Under dry heating conditions, corn protein hydrolysate could be easier to undergo the Maillard reaction	[78]

**Table 2:** An overview of CMRP applications.

Materials Reacted with Chitosan	Application Fields	Effect	References
Galactose	Food storage	Used as additives that improved the antioxidant quality of apple juice	[70]
Maltose	Food storage	Inhibited the discoloration and ascorbic acid content of fresh-cut Typha latifolia L.	[73]
Glucose	Health food or medicine	Exhibited higher anti-α-amylase and anti-α-glucosidase activities which could be used to treat Type 2 diabetes	[74]
Xylose	Food storage	Exhibited an extended shelf-life of semi-dried noodle for more than 7 days and limited the discoloration and darkening	[75]
Nisin	Health food or medicine	Exhibited anti-obesity effect by the alleviated microphage accumulation in mice liver and gut dysbiosis	[99]
Glucose	Food storage	Inhibited microbial growth and made the fresh goat sausages firmer and more stable	[100]
Glucose	Food storage	Inhibited lipid oxidation of fresh pork samples	[101]
Tropomyosin	Health food or medicine	Reduced the allergenicity of tropomyosin which is correlated with the reduction of α-helix and increase of β-sheet	[102]

## Data Availability

Not applicable.
